# Short-Term Detection of Dynamic Stress Levels in Exergaming with Wearables

**DOI:** 10.3390/s25216572

**Published:** 2025-10-25

**Authors:** Giulia Masi, Gianluca Amprimo, Irene Rechichi, Gabriella Olmo, Claudia Ferraris

**Affiliations:** 1 Department of Control and Computer Engineering, Politecnico di Torino, Corso Duca degli Abruzzi 24, 10129 Torino, Italy; giulia.masi@polito.it (G.M.); gianluca.amprimo@polito.it (G.A.); irene.rechichi@polito.it (I.R.); gabriella.olmo@polito.it (G.O.); 2 Institute of Electronics, Computer and Telecommunication Engineering (IEIIT), National Research Council (CNR), Corso Duca degli Abruzzi 24, 10129 Torino, Italy

**Keywords:** stress, exergames, rehabilitation, affective computing, EDA, HRV, wearable, biosignals

## Abstract

This study evaluates the feasibility of using a lightweight, off-the-shelf sensing system for short-term stress detection during exergaming. Most existing studies in stress detection compare rest and task conditions, providing limited insight into continuous stress dynamics, and there is no agreement on optimal sensor configurations. To address these limitations, we investigated dynamic stress responses induced by a cognitive–motor task designed to simulate rehabilitation-like scenarios. Twenty-three participants completed the experiment, providing electrodermal activity (EDA), blood volume pulse (BVP), self-report, and in-game data. Features extracted from physiological signals were analyzed statistically, and shallow machine learning classifiers were applied to discriminate among stress levels. EDA-based features reliably differentiated stress conditions, while BVP features showed less consistent behavior. The classification achieved an overall accuracy of 0.70 across four stress levels, with most errors between adjacent levels. Correlations between EDA dynamics and perceived stress scores suggested individual variability possibly linked to chronic stress. These results demonstrate the feasibility of low-cost, unobtrusive stress monitoring in interactive environments, supporting future applications of dynamic stress detection in rehabilitation and personalized health technologies.

## 1. Introduction

Stress detection through biosignals involves measuring physiological indicators–such as heart rate, skin conductance, and brain activity–to infer emotional and cognitive states through nervous system arousal. Recent advances in wearable technology and machine learning have enabled accurate, real-time analysis of physiological data for emotion monitoring. Arousal detection is critical in several domains, including mental health assessment, workload evaluation, user experience optimization, and rehabilitation.

In rehabilitation, the increasing adoption of digital technologies is transforming the intervention design by promoting long-term adherence and therapeutic effects through engagement and sustainability. In fact, digitalized cognitive and physical rehabilitation solutions represent a promising approach to remotely support the aging population and relieve healthcare systems, while improving users’ quality of life. Among digital solutions, exergames have gained popularity, as they combine physical and cognitive stimulation that can be adapted to individual needs [1,2,3]. The detection of players’ emotional and stress states during gameplay could enable real-time adaptations based on individual physiological responses [4]. For example, adaptive systems can modify the difficulty or content of the game in response to specific stress or excitement levels, creating a more engaging and customized experience [5]. In this context, emotional state measurement systems must be non-invasive, lightweight, and cost-effective in order to provide a serious, democratic solution for fair and widespread adoption. Wearable devices, especially wristbands equipped with physiological sensors, are increasingly used to monitor stress and emotional states during interactive and dynamic tasks [6]. Among the available biosignals, electrodermal activity (EDA) and blood volume pulse (BVP) are widely recognized indicators of autonomic arousal and can be unobtrusively measured with wearable devices, leading in the gaming context [7,8,9,10].

Despite technological progress, objective and continuous stress detection in ecological rehabilitation contexts remains challenging. Emotional assessment still largely relies on self-report questionnaires, highlighting the need for objective, continuous, and non-invasive monitoring tools [11,12]. Moreover, the optimal combination and positioning of sensors, as well as robust analysis methods for dynamic biosignal-based stress detection, are still under investigation [13]. Designing a protocol that is able to induce a specific emotional or stress response in subjects also poses difficulties, given the numerous variables to control and the potential inter-subject variability. These challenges are particularly exacerbated in uncontrolled, real-life scenarios [14]. Hence, many works in the literature have adopted simplified approaches that contrast a task, identified as high-arousal state, with rest periods, rather than measuring absolute stress levels. To date, only a few works have adopted real-time stress detection during dynamic tasks such as games [13,15].

Within this framework, this observational study assesses the feasibility of using an off-the-shelf wristband for short-term stress detection during the playing of a customized exergame with dynamic cognitive–motor demands. Twenty-three participants completed the four game levels, progressively increasing the intensity of the stimulus, while their physiological signals (EDA and BVP) were collected together with questionnaires and in-game performance metrics.

The primary objective of this study was to determine whether the proposed setup can reliably identify different levels of induced stress in a realistic exergaming scenario, with the long-term goal of supporting adaptive rehabilitative exergaming. In addition, the identification of the most informative features for stress detection aims to optimize the setup for future developments. Accordingly, this work addresses the following main research questions (RQ):

- RQ1: Can biosignals collected from an off-the-shelf wristband reliably discriminate between different levels of stress induced by a rapidly evolving exergame, and which signal features contribute most to this discrimination?
- RQ2: To what extent can this setup be used to accurately classify stress levels using machine learning methods?

Furthermore, this study explores potential confounding factors within the experimental protocol, addressing the following additional exploratory question:

- RQ3: Can factors such as prior stress levels or in-game performance influence physiological stress measurements?

## 2. Related Work

Stress detection through biosignals lies at the intersection of affective computing, physiology, and human–computer interaction. According to the literature, stress can be understood as a multifaceted psychophysiological state that arises when individuals perceive a discrepancy between external or internal demands and their resources to cope with them. Although often associated with negative experiences, stress is not exclusively maladaptive: it can manifest as distress when demands are perceived as overwhelming and harmful, or as eustress when challenges are appraised as motivating and growth-promoting. Beyond this appraisal-based perspective, stress is also commonly described as the body’s homeostatic response to external stimuli (stressors), encompassing physiological, cognitive, and emotional reactions that prepare the organism for action. Although stress is not classified as a discrete emotion, it is intrinsically linked to affective processes because it typically occurs under conditions of heightened arousal and negative valence within dimensional models of emotions [16,17]. This conceptual overlap allows stress to be viewed as an affective state situated at the intersection of emotional experience and workload. In fact, ‘stressed’, ‘distressed’, and ‘tense’ are some of the emotions pictured in the recent 2D and 3D models of emotions [18,19]. As a result, the study of stress shares methodological ground with emotion recognition research, since both rely on self-assessment techniques (e.g., questionnaires, subjective reports) and physiological signals (e.g., HRV, EDA) to infer internal states that cannot be directly observed.

Continuous measurement of autonomic nervous system activation enables a quantitative understanding of emotional and cognitive processes, which is essential for developing adaptive digital health and rehabilitation solutions. The shift from laboratory sensors to consumer wearable devices has democratized stress monitoring, supporting applications from entertainment to remote rehabilitation. The research field is rapidly evolving, and several open-access datasets are shedding light on lightweight biosignal-based stress detection. The CLAS dataset [20] includes electrocardiography (ECG), photoplethysmography (PPG), and EDA recordings with Shimmer sensors (Shimmer Research, Dublin, Ireland) of 62 subjects performing various cognitive stressing tasks. The WESAD dataset provides multimodal data (ECG, respiration, EDA) from 15 participants exposed to the Trier Social Stress Test using both Respiban (PLUX Wireless Biosignals S.A., Lisbon, Portugal) and Empatica E4 (Empatica Srl, Milano, Italy Italy) devices [21]. More recently, the dataset presented in [22] contains recordings from Muse S EEG headbands (InteraXom, Toronto, ON, Canada) and Empatica E4 wristbands of approximately 23 subjects performing multiple cognitive activities in both controlled and ecological settings [22]. Finally, Ometov A. et al. have recently reviewed open-access data in the field of stress and emotion in [16]. Looking at stress detection approaches, most studies in this field provide good results on contrasting resting and task conditions, rather than different levels of stress. However, the growing number of available data represents a great resource for developing and benchmarking generalized stress recognition pipelines. An example of a first effort towards generalized solutions is the work of Lili Zhu et al. [23], which tries to target the problem of detecting stress throughout EDA recordings from the wrist using multiple available datasets.

In gaming research, stress and emotional engagement have traditionally been assessed through subjective reports. Some comprehensive works by Kivikangas et al. [24], Yannakakis et al. [25], and Guthier B. et al. [26] underscored the growing role of biosignals in capturing player experiences. Commonly employed modalities include EDA, heart rate (HR) and heart rate variability (HRV), respiration rate, and skin temperature. The review of Welsh et al. [27] highlights that HRV is widely used to assess sympathetic activation in competitive gaming, but most studies rely on block-based comparisons (e.g., pre-game vs. in-game vs. post-game) rather than continuous, event-level monitoring. In addition, research indicates that classifiers for this task-based comparison of EDA and HRV signals are particularly effective, with many studies confirming their utility in virtual environments [9,10]. Similarly, Lazarou et al. [13] reviewed wearable stress prediction pipelines in laboratory and daily-life contexts, noting that ground-truth labeling is typically limited to baseline versus stressor phases or retrospective self-reports, without the continuous annotation that would be necessary in gameplay. Only a few works have demonstrated the feasibility of continuous monitoring. For example, Ishaque et al. [15] combined HRV, EDA, and respiration during VR simulations, extracting short-window features and applying tree-based models to classify stress; their personalized models achieved high accuracy (around 97%) in subject-dependent models, with lower results in subject-independent cases (mean of 65%). Together, these findings suggest that while biosignals are validated stress markers and are widely applied, relatively few studies have achieved continuous stress monitoring during games, despite their potential for adaptive gameplay, feedback, and therapeutic interventions.

Finally, to provide a solid foundation for our methodological approach, previous works describing the physiological basis and processing techniques of EDA and BVP signals are briefly summarized below, as these two biosignals are among the most widely used in stress detection research and are the focus of our work.

### 2.1. Electrodermal Activity

Electrodermal activity (EDA), also known as Galvanic Skin Response (GSR), is a widely used psychophysiological measure that captures variations in the electrical conductance of the skin, handled by the sympathetic branch of the autonomic nervous system. Hence, it offers a sensitive indicator of sympathetic arousal, reflecting a range of underlying processes, including emotional arousal, cognitive load, attention, and stress. EDA is usually described in terms of two primary components: the Skin Conductance Level (SCL), a slow-varying component, commonly referred to as the tonic component; and the Skin Conductance Response (SCR), a fast-varying signal, also referred to as the phasic component, which typically occurs 1 to 5 s after a stimulus, with amplitudes greater than 0.05 µS considered significant. Established processing approaches to retrieve signal components rely on continuous and discrete decomposition analysis or on a convex optimization problem, such as cvxEDA. A more comprehensive overview of methods is provided in [8]. Despite its sensitivity, EDA lacks specificity due to influences from habituation, anticipation, stimulus summation, and individual physiological states. In addition, the response may be influenced by other mental processes or intense physical activity. Nevertheless, its value lies in capturing continuous and unconscious physiological responses that overcome key limitations of self-reports. EDA has been extensively used in emotion detection, human–computer interaction, gaming, and more recently in digital health, where innovations in wearable sensors and signal processing have expanded its application in real-world contexts [28,29,30].

### 2.2. Blood Volume Pressure and Heart Rate Variability

**Figure 1:**
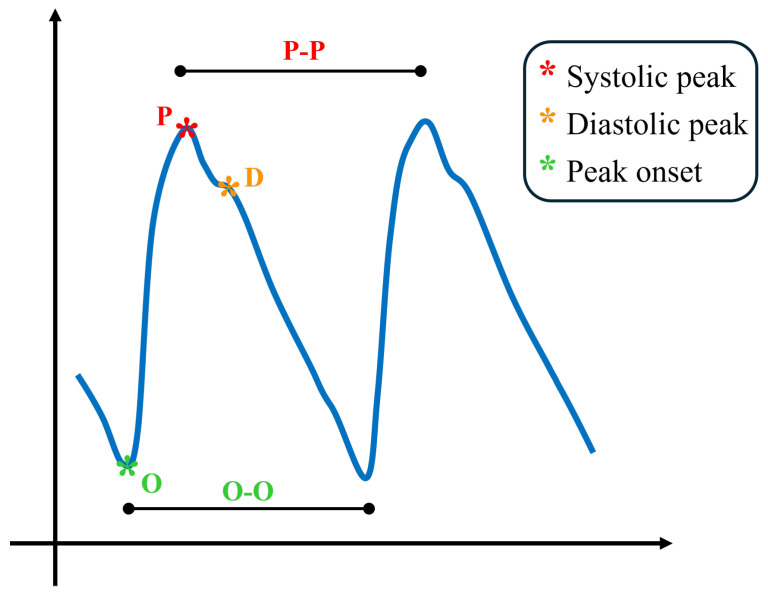
Qualitative blood volume pulse waveform representation with its main landmarks.

The blood volume pulse (BVP) signal waveform is primarily generated by the dynamic interplay between heart contractions and arterial wall properties, which results in changes in pressure and flow. This physiological phenomenon is known as the pulse wave, which can be observed and measured to analyze arterial elasticity and cardiovascular function. In Figure 1, a typical proximal pulse wave is shown, as well as some typical landmarks. The waveform contains several characteristic landmarks that reflect the underlying hemodynamic events. The onset of the waveform, denoted as O, represents the foot of the pulse wave and marks the beginning of the rise in arterial pressure. Following this, the systolic peak (P) emerges as the dominant feature of the cycle, generated by the forward-propagating pressure wave produced during ventricular ejection. After the systolic peak, a small inflection known as the dicrotic notch can often be observed, corresponding to aortic valve closure and the brief transient pressure change that it induces. Subsequently, the waveform may display a secondary feature, the diastolic peak (D), which arises from the reflection of the pressure wave in the peripheral vasculature. When the BVP signal is analyzed in conjunction with the ECG, the onset point O is consistently observed after the R-peak of the ECG. The time interval between these two points is usually referred to as the pulse arrival time (PAT), a parameter that reflects not only the vascular transit time of the pressure wave but also the pre-ejection period associated with the electromechanical delay of the heart [31,32].

The most common way of obtaining BVP non-invasively is PPG. PPG is an optical technique that measures changes in blood volume indirectly, detecting the variations in light absorption or reflection caused by pulsatile blood flow beneath the skin. BVP is now widespread outside of clinical practice, as PPG sensors can be easily embedded in personal devices, such as smartwatches. Moreover, from BVP, it is possible to retrieve information about HRV. HRV refers to a group of metrics that describe the variation in the rhythm of the heartbeat. These metrics were originally computed on the series of R-peaks in ECG. However, today, HRV is often measured from BVP using PPG technology, as this solution is more affordable and portable. HRV metrics are computed from BVP by first identifying the systolic peaks (P) in the signal. The series of time distances (intervals) between consecutive peaks then represent the HRV. The reciprocal of these intervals is known as the heart beat rate (from the ECG), which is equivalent to the pulse rate estimated from the PPG. Usually, intervals are given in ms, whereas rates are given in bpm. HRV metrics are now extremely popular in affective computing and fitness applications [7,33], as there is a plethora of automatic HRV analysis tools available for diverse applications [34].

## 3. Materials and Methods

**Figure 2:**
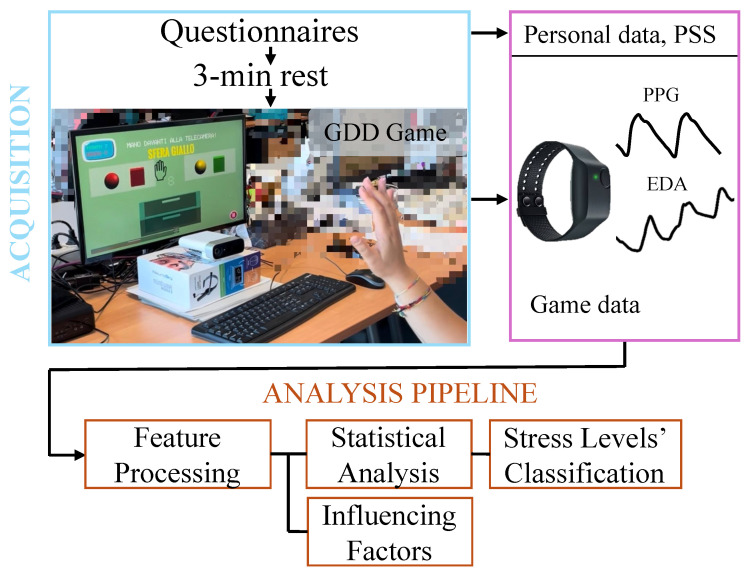
Schema of the data acquisition protocol and analysis pipeline, showing the experimental setup and the resulting collected data.

The primary objective of this work was to evaluate physiological arousal responses across a cognitive–motor task designed to induce different stress conditions. To this end, an observational study was designed, involving a data acquisition protocol and an offline data analysis pipeline. The experimental data included biosignals, specifically EDA and BVP, and surveys. A schematic overview of the acquisition protocol and data analysis pipeline is presented in Figure 2. The following subsections describe each element of the experimental protocol in detail.

### 3.1. Participants

A convenience sample of 40 healthy volunteers was recruited for the experimental session (mean 25 ± 4.5 years old, 28 males and 12 females, all holding a diploma, 74% holding at least a bachelor’s degree). Subjects were selected from among students and researchers from the Politecnico di Torino, and they received no compensation for their involvement. All procedures were carried out in accordance with the Declaration of Helsinki and approved by the Ethics Committee of the Hospital A.O.U. Città della Salute e della Scienza di Torino (Approval No. 00384/2020). Participants received comprehensive information about the purpose and execution of the study, including details on the instrumentation used, and provided written informed consent prior to participation. After the data acquisition phase, a final cohort of 23 subjects was included in the analysis, composed of 15 males and 8 females, in the age range between 20 and 30 years old (mean: 24 years), presenting with no mental or physical disorders and no need of medications.

### 3.2. Data Acquisition Protocol

Data acquisition closely followed the protocol described in [35]. Its visual schematic representation is shown in the upper part of Figure 2. Briefly, after providing demographic information, participants completed the Perceived Stress Scale (PSS) questionnaire [36], which assesses perceived stress levels over the past month. The Empatica E4 wristband was then placed on the wrist of the non-dominant hand, and the recording started. Each session began with a 3 min resting period with eyes closed to capture baseline physiological activity. Following this, the participants were given general instructions about the exergame objectives and the interaction method, without disclosure of specific challenges. Gameplay of the cognitive–motor task then occurred while the participants wore headphones. Each exergame session lasted approximately 3 min. More details on the design and characteristics of the game are reported in Section 3.3. At the end of the data acquisition protocol, the following data were collected for each subject:

- Personal information, such as age and gender;
- The PSS score;
- The physiological signals recorded with Empatica E4, including EDA and BVP signals, provided as CSV files containing Unix timestamps and sampling frequencies;
- Game data, including timestamps of game events, recorded errors, and scores.

### 3.3. The Task: The Grab–Drag–Drop Exergame

The cognitive–motor task employed in the study was the Grab–Drag–Drop (GDD) exergame, which was originally presented in [35]. This exergame is specifically designed to solicit players’ arousal by including a series of stressors of increasing strength across levels. The objective of the game is to repeatedly select the correct object from the four options displayed on the screen and drop it into the designed collection box. On-screen text instructions specify the objects to grab. Human–computer interaction is based on GMH-D, a low-cost hand-tracking solution based on computer vision and deep learning [37,38] that exploits an RGB-Depth camera (Azure Kinect, Microsoft Corporation, Redmond, Washington, USA). GMH-D allows hands-free interaction with the game, while the game algorithm recognizes the *grasp* and the *open-hand* gestures from the joints’ trajectories, allowing the player to grasp, drag, and release (drop) the objects in the scene.

The game includes a simple reward mechanism based on points and errors. If the player grasps the wrong object, drops it outside the collecting box, or the time runs out, an error is assigned; otherwise, one point is scored. Errors and points are associated with negative and positive acoustic feedback, respectively. As mentioned, the levels grow in complexity and the music tempo is increased in every level. A short description of the levels follows:

- Level 1: Tutorial level; six selections focused only on color differentiation among cubes; 10 s time limit per selection; normal-paced background music.
- Level 2: Adds variety in objects’ shapes (cube, sphere, cone, cylinder); colors and shapes can repeat, requiring identification of the correct shape–color combination.
- Level 3: Introduces Stroop-test-like [39] interference with mismatched text–color instructions; time per selection reduced to 6 s.
- Level 4: Most challenging level, includes all previous features plus a moving collection box; still a 6 s limit per selection.

Level transitions are seamless, without explicit notifications, to maintain the user in a flow state, i.e., a high-engagement condition. Game log data generated during the gameplay (e.g., game events, timings, and performance) are stored in JSON format for subsequent offline analysis. The game integrates motor rehabilitation (hand dexterity and coordination tasks) and cognitive challenges (stressors and distractions), designed primarily for research on workload and stress measurement.

### 3.4. Data Analysis Pipeline

#### 3.4.1. EDA Processing and Feature Extraction

**Figure 3:**
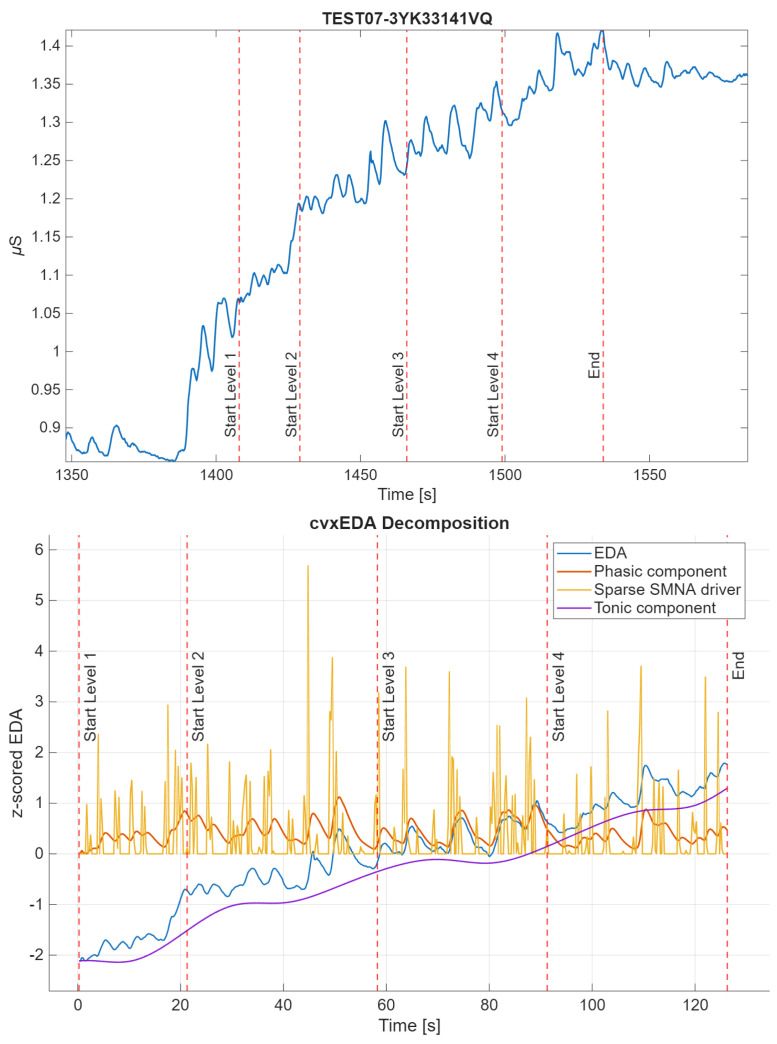
Example of the windowed z-scored original signal of subject 15 and its decomposition through cvxEDA algorithm [41]. Start and stop of the game levels are shown as vertical lines. The cvxEDA algorithm provides the tonic component (*t*), the phasic component (*r*) and the sparse sudomotor nerve activity (SMNA) driver of phasic component (*p*). The cvx model is physiologically inspired and directly deal with the uncontrolled inter-stimulus interval typical on in-the-wild recordings.

EDA analysis and feature extraction were performed in MATLAB R2023. There is no gold-standard method for EDA analysis in the wild, so multiple well-established techniques were used. The raw EDA signal was first low-pass filtered (using a Butterworth filter, first-order) with a cut-off frequency of 1.5 Hz, as in [40]. The signal was then windowed for the duration of the experimental protocol, including the 4 game levels and the initial rest phase. The resulting windowed signal was z-scored (referred to as EDA) and decomposed into tonic (cvxT) and phasic (cvxP) components through the convex optimization algorithm cvxEDA [41]. An example of signal decomposition is shown in Figure 3. Features were extracted in each game level, in the time and non-linear domains. Frequency-domain features were computed but were only used for comparisons across averaged game levels. They were excluded from within-level comparisons because the game levels varied in duration, both across levels and subjects, and were generally too short, introducing potential spectral biases. In particular, the frequency resolution and bands required for EDA analysis could not be achieved for the fastest subjects.

Statistical descriptors for EDA, cvxT, and cvxP were computed for each game level, including the mean, median, standard deviation, range, maximum, skewness, and kurtosis. Additional features were computed for EDA. Non-linear dynamics were characterized via Shannon entropy, approximate entropy, sample entropy, and the largest Lyapunov exponent for EDA. In addition, Hjorth features (activity and mobility) [42] were calculated, even though they are more often used in EEG analysis, as suggested by [30].

Lastly, the first derivative of the EDA signal was also calculated to summarize the EDA trend. In particular, the mean absolute derivative, the maximum absolute derivative, and the mean negative derivative were calculated, as reported in [30]. Table 1 shows a summary of the extracted features.

#### 3.4.2. BVP Processing and Feature Extraction

The Python HeartPy module (in the Python Heart Rate Analysis Toolkit) was used to extract features in each game level. HeartPy provides the peaks’ location, the PP distance series, and an automatic outlier identification and rejection tool. Thus, the toolbox automatically retrieves the tachogram—i.e., the series of the peak-to-peak distances PP—and then it provides a set of standard HRV analysis features. The features belong to the time domain; the breathing rate is also estimated. The detailed list of computed features and the methodology applied can be found in the module documentation [43]. In addition, the original computed tachogram was further processed, retaining only $pp_{i}|Perc\left(5\right)<pp_{i}<Perc\left(95\right)$. From the resulting time series, some additional features were then computed—specifically, the mean PP interval, the root-mean-square of successive differences (rmssd), the standard deviation of the PP series (sdpp), and the interquartile of the PP distribution (iqr).

**Figure 4:**
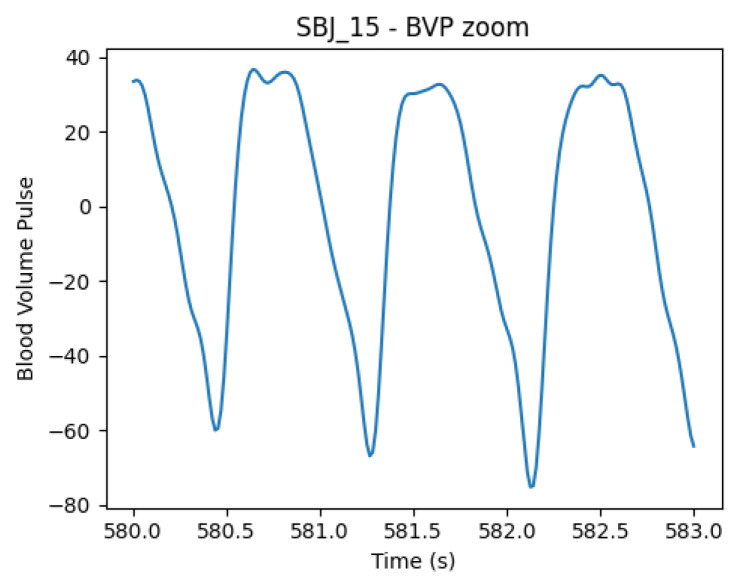
BVP signal from subject 15, showing systolic and diastolic peaks with unusual shape and similar amplitudes, causing inaccuracies in peak positions’ detection. The phenomenon can be caused by the positioning of the wristband and pressure caused by wrist movement, as shown in [44].

On top of that, due to the presence of a non-standard shape in the signals (shown in Figure 4), leading to a faulty behavior in the peak P detector algorithm, the same feature extraction was also performed on the inverted signal. In this configuration, the HeartPy feature extraction pipeline was fed with inverted signals and identified the onset peaks (now positive); hence, the obtained series consists of OO distances instead of PP distances (see Figure 1 for landmark definition). As already mentioned, the deformation of the PPG signal with this type of sensor is not uncommon; in fact, the modality of the PPG recording, especially location and pressure, influences the shape of the waveform [44]. These aspects sometimes challenge traditional automatic algorithms for identifying systolic peaks (P), causing ostensible variability between successive beats. The use of the O landmarks instead of the P peaks in the estimation of HRV features is not completely new to the literature. For example, Singstad B. et al. [45] tried different points to compute HRV metrics, finding the OO interval reliable in rest conditions. However, the consensus on best practice and implications is not final.

#### 3.4.3. Feature Statistical Analysis

The analysis was first focused on the study of the arousal response obtained with the different stimuli of the 4 game levels. Descriptive statistical analysis in terms of violin plots, box plots, and normality tests was performed through Jamovi 2.2.5 [46] and custom scripts in Python 3.11.7. Due to intrinsic feature redundancy and dataset dimensionality, in order to understand the behavior and significance of the extracted features and to create meaningful graphical representation, a subset of 20 features was pre-selected from the 95 computed. First, the features were divided into clusters based on reciprocal cross-correlation. A feature was assigned to a cluster when it had a significant Spearman coefficient greater than 0.8 with any of the features of the cluster. For each cluster, only the feature with a higher correlation coefficient with the game level label was kept. Exploratory inference statistical analysis was also performed in terms of within-subjects (repeated-measures) ANOVA and post hoc pairwise *t*-test. In this way, it is possible to understand whether there is a significant effect of the game level on the feature. In case of non-normality of the features (Shapiro–Wilk *p*-value < 0.05), the Friedman non-parametric alternative and pairwise Wilcoxon signed-rank tests, as post hoc pairwise comparison tests, were considered. The Python pingouin package was employed for this analysis.

After this procedure, the features highlighted from the statistical analysis were used to create 3 final datasets of 3, 5, and 7 features that were considered for further analysis.

#### 3.4.4. Classification of Stress Levels

A subject-level machine learning pipeline was developed in Python to evaluate the classification performance of multiple algorithms on the multi-class level problem. The goal was to further demonstrate that the computed features could be used to robustly characterize different arousal conditions, as also supported by conventional statistical analysis, rather than obtaining an unbiased classifier. Indeed, the latter would require a much larger dataset than the one currently available. The final dataset used for classification consisted of 92 samples derived from the 23 subjects, with 4 samples per subject (1 for each game level). The identification IDs were used to identify individual subjects and to define the folds for cross-validation.

The three feature sets (with the top 3, 5, and 7 features, respectively) obtained from the prior illustrated feature selection process were tested. For each feature set, the classification pipeline was executed independently.

The pipeline employed an outer Leave-One-Subject-Out (LOSO) cross-validation strategy, where in each iteration, the data from one subject were held out as the test set, and the remaining data from the other twenty-two subjects were used for training. Within each outer fold, a nested 3-fold stratified cross-validation was used to optimize the model hyperparameters via Bayesian optimization, implemented with BayesSearchCV from the scikit-optimize Python library.

Four classifiers were evaluated: Support Vector Machine (SVM), Random Forest (RF), k-Nearest Neighbors (kNN), and XGBoost. Feature scaling was applied using standardization for the SVM and kNN models only, while the RF and XGBoost models were used without scaling.

To ensure the robustness and reproducibility of the results, the entire pipeline was executed using five different fixed random seeds. For each configuration (i.e., feature set and seed), the performance was evaluated by averaging the classification results over all 23 LOSO iterations. The performance metrics reported included accuracy, precision, recall, F1-score, and the area under the ROC curve, computed using a one-vs.-rest strategy to accommodate the multi-class nature of the problem. Finally, Shapley (SHAP) values were obtained from all models to evaluate the relevance of the features. The model-agnostic kernel explainer was used for SHAP computation in the SVM and KNN models, while the model-specific kernel explainer was used for tree-based models.

#### 3.4.5. Influencing Factors

In order to have a broader understanding of the collected data, the effects of in-game performance and PSS scores were analyzed.

In-game performance was computed as points per second using the formula $Performance_{Score}=\left(Points-Errors\right)/LevelDuration$, where Points corresponds to the number of objects correctly dropped into the collection box, while Errors represents the number of objects missed.

To evaluate whether the subject’s performance in each level influences the value of the extracted physiological features, a series of Linear Mixed Models (LMMs) were performed. This approach was chosen to appropriately account for the repeated-measures nature of the data (multiple tasks per subject) and the nesting of observations within subjects, allowing for the estimation of both fixed effects (common to all subjects) and random effects (individual subject variability).

For each physiological feature, a separate LMM was constructed with the following structure: Feature∼Game(LABEL)+Performance+(1|SubjectID). In this model, the following applied:

- The feature (e.g., specific EDA or BVP feature) was the dependent variable.
- Game(LABEL) was included as a fixed-effect categorical predictor to assess whether the mean feature values differed significantly across the four tasks.
- Performance was included as a fixed-effect continuous predictor to determine its linear influence on the feature.
- (1|SubjectID) specified a random intercept for SubjectID, accounting for baseline individual differences in feature values that were not explained by Game or Performance. This allowed each subject to have their own unique intercept while allowing for task-specific performance effects.

Given that the models were run for a large number of features, multiple comparison corrections were applied to the *p*-values obtained for the Performance fixed effect. Both the Bonferroni and the Benjamini–Hochberg false discovery rate (fdr) corrections were computed. Due to the small sample size, the models were kept as simple as possible, ignoring possible interaction effects and keeping only a random intercept, not slope. Moreover, the features were pruned upfront considering the cluster of correlated features described in Section 3.4.3, this time considering the correlation with performance instead of the label. In addition, the correlations of the features with the performance values were considered for comparison.

Moreover, the general stress condition, evaluated through the PSS score collected from each subject before the trial, was investigated as a possible influencing factor. Since the PSS score was available only per subject, the analysis was therefore performed at the subject level. Performance and physiological features (EDA and BVP) were averaged across the four game levels to obtain a single value per subject, and their correlation with the PSS scores was then explored.

## 4. Results

As stated in the Methods section, we collected data from 40 participants. However, when the data were analyzed for results production, it was necessary to reduce the cohort of participants. Data from 17 participants had to be excluded due to technical issues, leaving a final cohort of 23 subjects for analysis. Specifically, in four cases, the Empatica E4 recordings were unusable due to human errors (incorrect sensor positioning or synchronization). In an additional 13 cases, the EDA signal exhibited an atypical pattern similar to low-amplitude random noise. Although no standardized criterion exists for an acceptable wrist-based EDA signal-to-noise ratio (SNR), a simplified approach considers the ratio of spectral power in the 0–0.5 Hz band, associated with sympathetic regulation [8], to that in the 0.5–2 Hz band. Accordingly, in addition to visual inspection, signals with an SNR below 20 dB were discarded. In contrast, the 23 retained signals showed SNR higher than 30 dB. Furthermore, signal variance was assessed, and signals were excluded when the variance fell below 0.001 μS^2^. The signals in the final dataset showed an average variance approximately ten times higher than this threshold. Although validating the Empatica E4 device for EDA acquisition and defining best practices for its use are beyond the scope of this study, the technical issue was traced back to suboptimal coupling between the Empatica E4’s sensors and the skin.

### 4.1. Features’ Statistical Analysis

After the exclusion of cross-correlated features (showing cross-correlation ranging from 0.882 to 0.998), a subset of 20 features was considered for the analysis. The descriptive statistics and box plots of the features were checked for visual inspection of abnormal values or significant outliers.

Among the 20 features extracted and selected, 7 were significant in the within-subject analysis; they are reported in Table 2. The Shapiro–Wilk test *p*-values were employed to check the normality of the distribution of the features in the four levels. Almost all 20 features, except for $oo_{breathingrate}$, $pp_{pnn20}$, and $pp_{sd1/sd2}$, had a non normal distribution (*p*-values < 0.01) in at least one level and were subsequently treated with non-parametric tests. The results of the statistical analyses, including repeated-measures ANOVA or Friedman tests, are summarized in Table 2. For the ANOVA, the reported metrics include the F statistic, the generalized eta-squared (ng2) as an effect size measure, and the corresponding *p*-values. For the Friedman test, the table reports the Friedman statistic and Kendall’s W, used as effect size indicators. Post hoc analyses with Bonferroni correction are also reported, specifying the number of significant pairs and their combinations.

**Figure 5:**
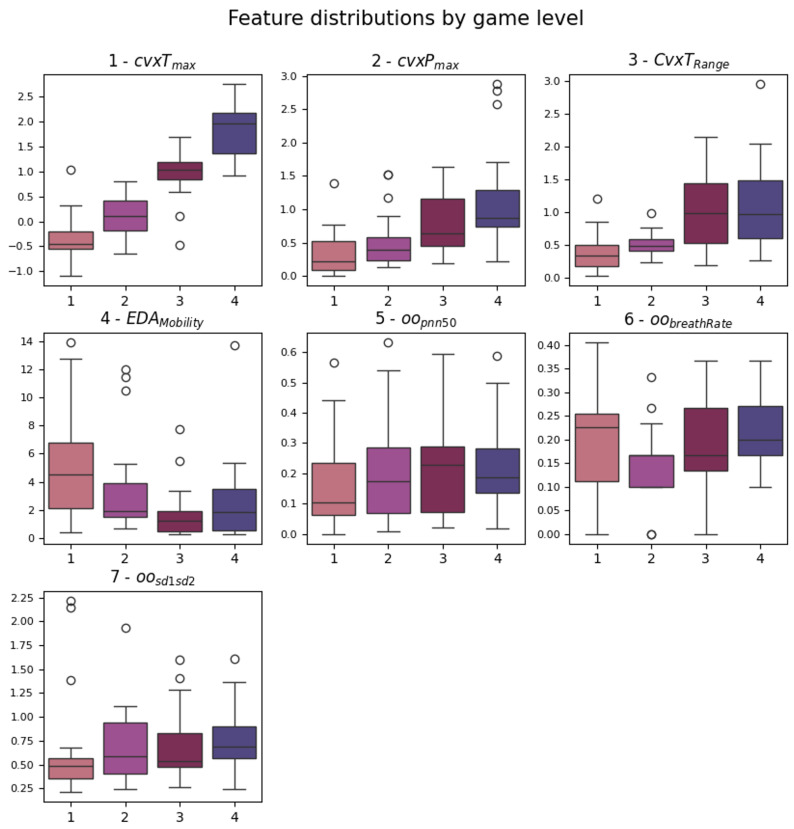
Box plots of the features that emerged from the statistical analysis. The significant pairs can be looked up in Table 2. The order of plotting follows the ranking considered to obtain the feature sets.

The $cvxT_{Max}$ and $cvxP_{Max}$ present significant *p*-values for each level comparison. They represent the maximum amplitude found in the tonic and phasic components of the EDA signal, as extracted by the cvxEDA algorithm. This means that both the fast peaks of activity and the slow average change of the signals differed during the four stimulation levels. This can also be said for $cvxT_{Range}$. Box plots of the identified features are shown in Figure 5. It is possible to note that $cvxT_{Max}$ increases monotonically with the game levels, while $cvxP_{Max}$, even if significant for each pair, has a higher variability in level 1. Regarding HRV metrics, they seem to be less affected by the gameplay; only features from the OO series (definition in Figure 1 and Section 3.4.2) emerged from the analysis, and $oo_{pnn50}$ significantly increased between levels 1 and 3/4. This feature is a standard HRV descriptor that indicates the percentage of consecutive intervals with more than 50 ms difference.

Since the statistical analysis of the features highlighted a small subset of seven features, they were directly used as a dataset for classification. They were also ranked in relevance considering the number of significant post hoc pairs, correlation to the label, and redundancy, identifying the top three and top five features to use as datasets for classifiers. The final order is the same as mentioned and used in Figure 5.

### 4.2. Classification of Stress Levels

**Figure 6:**
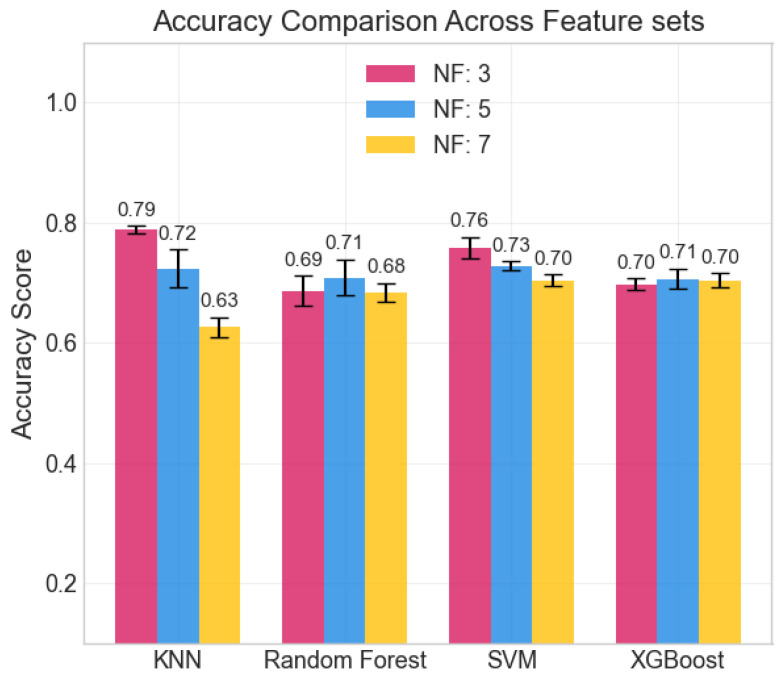
Averaged accuracy scores across the 5 seeds. Error bars show standard deviation.

**Figure 7:**
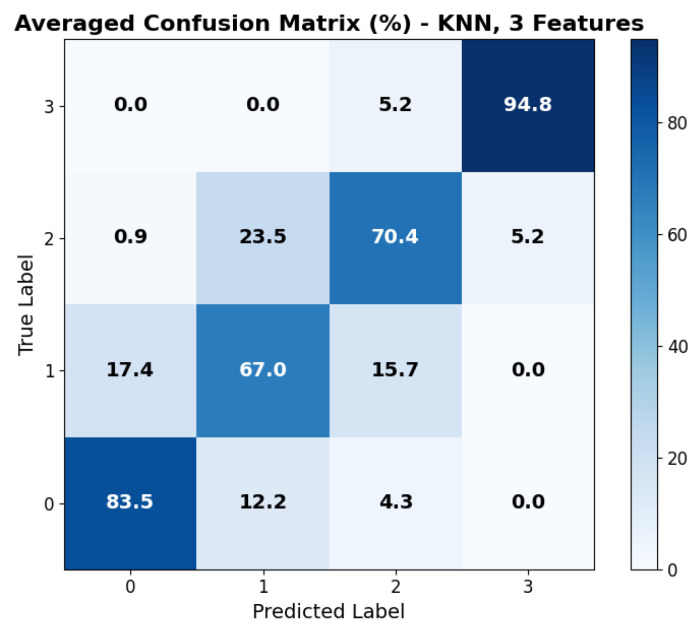
Averaged confusion matrix across the 5 seeds for the best model: KNN with feature set 3. Labels 0 to 3 refer to game levels 1 to 4.

The repeated LOSO cross-validation provided good results. All metrics (accuracy, precision, recall, and F1-score) scored above 0.6 for all classifiers in all configurations. KNN provided the best accuracy, recall, and precision (all above 0.78) with the set of three features: $cvxT_{Max}$, $cvxP_{Max}$, and $cvxT_{Range}$. Its performance, however, was less stable than that provided by tree-based methods (RF, XGBoost) when changing the feature set size. SVM shows similar behavior across feature sets. The averaged accuracy scores across the five seeds are reported in Figure 6. The confusion matrix, averaged across the five seeds of the best model (KNN), is shown in Figure 7. It clearly shows that level 4, corresponding to the highest stress condition, is identified with great accuracy, never confused with level 1. In addition, the main source of misclassification is the confusion of intermediate and adjacent levels (1 with 2; 2 with 3).

**Figure 8:**
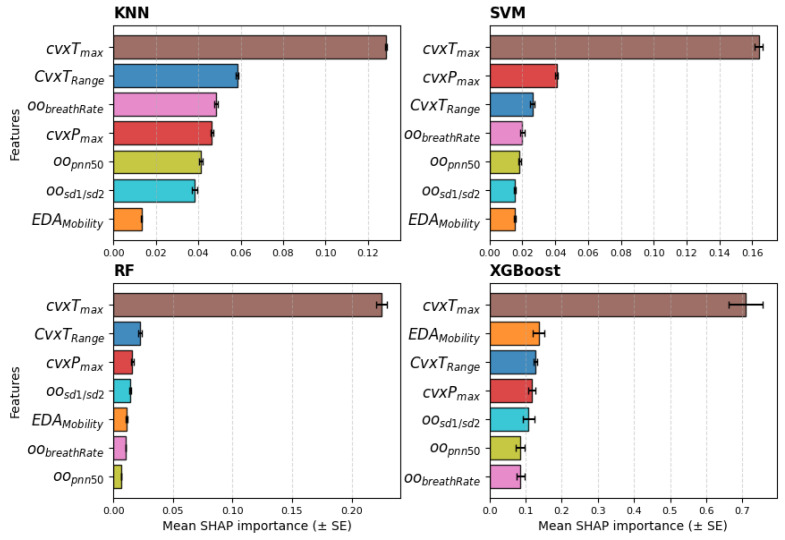
Relevance ranking of the features obtained with SHAP values for all models averaged across the 5 seeds; feature set = 7. The bars show the mean and standard error (SE).

Lastly, the relevance ranking, obtained from the SHAP values averaged across the five seeds of the models, when trained with the seven-feature dataset, is reported in Figure 8. The feature $cvxT_{max}$ clearly emerges as the most relevant, while the other features show no distinct differences in relative importance. The SVM and RF models show the same top three features as those selected by the statistical analysis.

### 4.3. Influencing Factors

Performance scores were generally lower in the first and last levels of the game, ranging from 0 to 0.333 points per second between subjects and levels. For the analysis of performance, the 95 features computed were pre-selected, as illustrated in the Methods section, selecting the most correlated to the performance for each feature cluster. The frequency features were included in the analysis of the influencing factors.

The analysis of the possible linear effect of game level performance on features was performed by LMMs. For reporting, features were identified based on an uncorrected *p*-value threshold of p<0.05 for the Performance effect. For these identified features, their uncorrected *p*-values (p_unc), and their Bonferroni (p_Bonf) and FDR-corrected (p_fdr) *p*-values are reported in Table 3. The significance of the effect does not withhold the correction factors; hence, the observations are qualitative. The correlation between the features highlighted by the LMMs and the performance in each level was computed to strengthen the results. $EDA_{Range}$ is the only feature that also shows a modest (−0.29) significant correlation (*p* < 0.05) with performance, in line with the LMMs’ results, showing a potential relationship. As no significance was found, no further conclusions can be derived for the BVP features. The coefficient for $EDA_{PB5}$ (power percentage in the 0.05–0.5 Hz band) could suggest a strong effect relative to its range; however, it is not significant, and the frequency feature could be unexpectedly biased, as discussed previously.

In addition, analysis at the subject level was performed, in order to better understand the impact of performance and PSS on the features. Hence, the features were averaged in the game levels to have one value per subject. Some features exhibited a significant correlation coefficient with the average game performance, as illustrated in Table 4. $pp_{breathRate}$, the breathing rate obtained indirectly from the BVP signal, seemed to be significantly correlated with performance, and not with level difficulty, and the PSS score. However, both $pp_{breathRate}$ and $cvxP_{PB5}$, even if they are averaged across levels, still present a notable correlation with the mean duration of the game levels (−0.4 with *p* > 0.05 and −0.57 with *p* < 0.01, respectively).

Regarding the PSS score, it ranged from a minimum of 6 to a maximum of 26 among the 23 participants. These values indicate that no subject scored a very high baseline stress level (scores in the range of 27–40 indicate a high stress level). Ten subjects scored less than 13, which corresponds to a low stress threshold, and thirteen subjects scored between 14 and 26, corresponding to moderate stress. The PSS score was found not to be correlated with any of the metadata, such as game duration or performance. In contrast, some features (averaged across levels) presented a significant correlation with the PSS score. There were no significant correlations for the BVP features, while many of the EDA features presented coefficients greater than 0.5. They are shown in Table 5.

**Figure 9:**
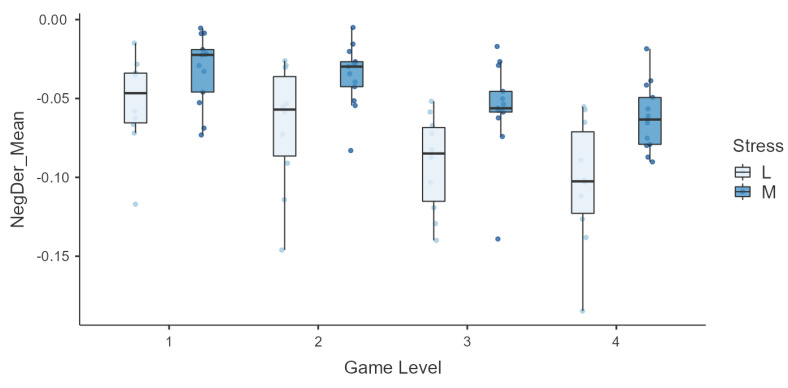
Box plots of the mean negative derivative of the EDA signal across game levels in the two groups ‘low stress’ (L) and ‘moderate stress’ (M), as determined by PSS scores.

Finally, we conducted an in-depth analysis for $NegDer_{Mean}$ due to its significance in the description of the subjects’ sympathetic nervous system response. $NegDer_{Mean}$ is the average of the negative values of the derivative of the EDA signal, so it represents the mean decrease velocity. It exhibits quite a high correlation with the PSS score, so it was also further explored across levels. In order to represent the variation in $NegDer_{Mean}$, subjects were aggregated in groups according to the PSS score with low and moderate baseline stress, and box plots across levels for the two groups are shown in Figure 9. The feature shows an interesting behavior with respect to the PSS score group, clearly presenting a lower rate of decrease in the signal for the ‘moderate’ group in higher levels.

## 5. Discussion

This section discusses the experimental findings in relation to the research questions identified in the Introduction section. Specifically, we evaluate whether biosignals collected from a wrist-worn device can discriminate different stress levels during gameplay (RQ1), how accurately such signals can classify stress levels using machine learning methods (RQ2), and whether individual factors, such as performance and perceived stress, can influence physiological responses (RQ3).

The analysis of the EDA signal and the derived features across the game levels of the Grab–Drag–Drop game confirmed that 23 subjects showed an arousal response. This finding suggests that the game can induce autonomic nervous system activation that is detectable through a lightweight setup, provided that proper skin–sensor coupling is established. With the support of the results of the paper presenting the game [35], we can, therefore, conclude that the game provides gradual stimulation to subjects, as intended by its design. The analysis pipeline highlighted significant features from the EDA signal, able to distinguish among four different stress levels with an accuracy up to 70%, and even higher for a two-level classification. These findings, discussed in detail in the following subsection, support the feasibility of using the Empatica E4, an off-the-shelf wearable device, to monitor dynamic stress levels during a rapidly evolving exergame, thereby answering the main research question of this study. Furthermore, the results suggest that the optimal setup could rely on the EDA signal alone, as it provides good results in this task, but reliable skin–sensor coupling should be guaranteed. Finally, regarding the exploratory research question on potential influencing factors, the Perceived Stress Scale correlated significantly with several EDA features. In particular, the $NegDer_{Mean}$ feature seems to be a promising low-cost objective marker for chronic stress.

### 5.1. Features’ Statistical Analysis

To address RQ1, we first examined whether biosignals captured by the Empatica E4 could reliably discriminate between different stress levels during the game. The statistical analysis of EDA and HRV features revealed several physiological patterns consistent with gradual activation across the game levels. As described in Section 3.4.3, the analysis showed promising differences in mean values across the game levels for 7 out of a subset of 20 features. This finding strongly supports the ability to characterize nuanced levels of activation during the task, not merely to distinguish them from a resting condition. Specifically, the EDA features provide statistically significant differences across all pairs of game levels.

The contribution of the slow tonic activity (cvxT) across the game levels seems to stand out as a discriminator. The behavior of the tonic and phasic components during the game levels suggests that the activation increases monotonically for both the slow component and peak amplitudes. From this perspective, the higher variability observed in $cvxP_{max}$ during the first game level could be interpreted as the phasic activation owed to the novelty of the task—see Figure 5. This hypothesis is supported by the $EDA_{Mobility}$ feature, which is defined as the square root of the variance of the first derivative of the signal divided by the variance of the signal itself, and it can be interpreted as the rate of change in the signal. Looking again at Figure 5, $EDA_{Mobility}$ is higher for levels 1 and 4, suggesting a greater compensatory response to the external stimulus.

In contrast, HRV metrics appear to be less affected by the gameplay. Only features from the OO series (see again Figure 1) emerged as relevant from the analysis. At first glance, the increase in $oo_{pnn50}$ between level 1 and levels 3/4 seems contradictory. In fact, over long time periods (e.g., above 5 min), this metric is usually expected to increase in relaxation conditions. However, the short-term behavior is less explored and may reflect a short-term (i.e., rapid) adaptation to the stimulus. Furthermore, the complex regulatory mechanism of the heart, which involves the parasympathetic intervention (absent in EDA), could prevent the detectability of certain physiological responses. This is plausible in our analysis, which calculated metrics over short windows corresponding to level durations (approximately 30 s). Alternatively, it could be that the game does not provide a stimulus that elicits this type of response. Despite the great attention in the literature, HRV features computed over short-term epochs have led to contradictory or inconclusive results across several studies, suggesting that the complexity of this topic has not yet been fully grasped. In general, the predictive performance of HRV metrics has not met expectations. For example, the work of Seungjae et al. [47] achieved very good results in rest vs. task classification, even over ultra-short-term epochs (1–2–3 min), using a commercial chest strap. On top of this, the fact that the emerged features at game level come from the OO series suggests that estimating heart rate from the onset peak (O), rather than the systolic peak (P), is more reliable for short-term HRV analysis from BVP collected ’in the wild’. This may be the consequence of increased systolic peak detection errors, suggesting the onset peak as a more robust alternative in the presence of non-standard signal shapes.

### 5.2. Classification of Stress Levels

To address RQ2, the extracted features were used as input for several classifiers to quantify the ability of biosignals to predict stress levels. The effectiveness of the features extracted with the proposed framework using a wearable device is supported by the results of the LOSO cross-validation, which achieved good accuracy across all classes. Among all features, $cvxT_{Max}$ consistently emerges as the most informative, as highlighted by statistical analysis, feature relevance rankings, and the performance of the KNN and SVM classifiers when varying the number of features. Specifically, the performance of both classifiers declined as additional features were included, underscoring the dominant contribution of $cvxT_{Max}$. The SHAP values from the models, shown in Figure 8, mostly confirm the selection of the top three features; however, apart from $cvxT_{Max}$, the remaining features exhibit similar relevance across all models. This suggests that features like $EDA_{Range}$ and $cvxP_{max}$, although statistically discriminative, do not provide substantial additional information beyond that already captured by $cvxT_{Max}$, despite their low cross-correlation with it.

The achieved average accuracy of around 0.7 in classification across four stress levels is consistent with findings in the literature [21,23,48,49]. Furthermore, the confusion matrix shown in Figure 7 clearly suggests that the selected features can distinguish between high and low activation levels: the main classification errors occur between adjacent classes, with no confusion observed between levels 1 (lower stress) and 4 (higher stress). While most studies in the literature focused on binary classification (e.g., rest vs. stressing task) [48], our multi-class results are consistent with previous findings. In particular, Zhu L. et al., in [23], evaluated wrist-worn devices across four different datasets, obtaining accuracies ranging from 0.68 to 0.92 in the binary classification. Moreover, the authors of the paper presenting the WESAD dataset [21] reported accuracies for a three-class classification problem (rest vs. amusement vs. stress). They achieved the following maximum accuracies: 0.70 from wrist BVP, 0.62 from wrist EDA, and 0.75 using all wrist-based sensors. Similarly, Greco et al. [49] achieved a balanced accuracy of 0.75 in a multi-class problem (identifying three different stress-inducing tasks) with features extracted from EDA sampled at 250 Hz. Finally, the study closest to our setup is that of Ishaque et al. [15], who extracted short-window features from HRV, EDA, and respiration during a VR game, managing to identify stress with a 0.65 accuracy for the subject-independent models.

### 5.3. Influencing Factors

Finally, to explore RQ3, we investigated whether individual factors modulated the observed physiological responses. This analysis aimed to identify potential confounding or mediating effects that could inform future experimental design.

In fact, the phenomenon of stress is complex and was found to depend on many internal or external factors. Regarding EDA, the normalization performed z-scoring of the signal, from rest to the maximum solicitation, accounting for baseline differences between subjects. However, there is no way of completely controlling the subjects’ experience, due to their internal processes. Among the multiple factors that could play a relevant role in our setting, we considered two in particular: the game performance, as a possible cause of different subjects’ reactions; and the PSS score obtained from subjects before the gameplay. In fact, it was found that providing feedback on the subjects’ in-game performance, like displaying errors and points, can boost focus and engagement but may also induce anxiety in cases of repeated failure (errors). Hence, we computed a performance score based on points and errors per second, in order to normalize data across the different lengths of the game levels.

The analysis conducted on in-game performance offered limited insights. The LMMs explored the relationship between performance and physiological features, with awareness of the game level. However, the LMMs’ coefficients for performance were not significant after statistical correction, even when keeping the model as simple as possible. $EDA_{Range}$ presented a significant modest negative correlation with performance, a finding consistent with the LMM results. This implies that when there is a better performance, the EDA signal span is less, which could be the result of fewer sudomotor nerve activity (SMNA) bursts, i.e., lower sympathetic nervous system activation. Finally, when analyzing the game levels as a whole—hence, averaging features and performance across all levels—we found a significant positive correlation (>0.4) for $pp_{BreathRate}$ and a greater correlation (>0.5) for $cvxP_{PB5}$.

Moreover, the general stress level of a subject, as measured by the PSS, is also expected to influence their physiological state and responses. Regarding the PSS score, a high significant correlation (>0.5) was found with multiple EDA features. In addition, Figure 9 suggests that $NegDer_{Mean}$, the EDA signal’s average speed of decrease, was faster for individuals who reported lower stress levels, particularly during the most challenging game levels (higher stimulation). Hence, subjects with higher levels of stress seem to maintain a longer sympathetic activation, requiring more time to return to baseline. This phenomenon could be a manifestation of suboptimal allostasis due to chronic stress [50,51] and could suggest the role of this feature as a potential biomarker of baseline stress. However, further research is required to support this hypothesis.

### 5.4. Limitations and Further Work

The reduction in the cohort from 40 to 23 subjects, due to technical issues in the sensor–skin coupling, prevented the extraction of results from a larger group. However, this limitation does not diminish the feasibility of the proposed lightweight setup, the methodological analysis, or the preliminary results. The skin–sensor coupling issues identified in this study should be further investigated in future work, potentially by considering wearable sensors that record the EDA signal from the palm rather than the wrist. Furthermore, most EDA data in the literature were collected from hands or feet; this approach could make it easier to compare methodologies and results with existing studies.

The proposed protocol was mainly exploratory of the feasibility of using a lightweight setup to estimate nuanced levels of instantaneous stress in a real-life application. This goal was fully achieved, as the extracted features yielded good classification performance. In addition, our experiment explored potential influencing factors on the recorded signals, such as the baseline stress level of the subjects. However, it is important to note that the protocol was not structured to prove clinical causation regarding stress. In fact, the real-life condition tested intrinsically introduces uncontrolled factors that could diminish the clinical power of the observations. First of all, the game level duration and auditory stimuli depend on the performance of the user; thus, these aspects are not consistent across all subjects tested. Secondly, the lightweight setup could be more susceptible to noise than the gold-standard instrumentation employed in clinical stress studies. Hence, when examining influencing factors, it is not possible to exclude potential biases introduced by the low-cost instrumentation and the partially uncontrolled setup. Lastly, the game includes different types of stimulation, providing an ecological experience, but the subject’s specific susceptibility to each kind of stimulation (e.g., rhythm and auditory sensitivity) can vary. For this reason, further studies in controlled environments are required to clinically prove the underlying relationship between baseline stress and autonomic nervous system responses. A deeper theoretical understanding of this relationship could pave the way for more reliable technologies for the automatic assessment of the general stress level of an individual.

Moreover, fostering interdisciplinarity is crucial, as the role of affect and emotion is often overlooked in many medical disciplines. First of all, the evaluation of the autonomic response, which determines the arousal response, remains understudied. Current assessments are often limited to clinical autonomic function tests for specific pathologies like dementia or Parkinson’s disease [52,53]. These are examples of pathological populations that could benefit the most from the adoption of remote rehabilitative solutions. Hence, it is not clear how these dysfunctions influence the autonomic response and the affective sphere, and it remains to be determined whether the conclusions obtained on healthy subjects can also be considered valid for and generalized to these pathologies.

Finally, the limited sample size included in the analysis (23 out of 40) may represent a shortcoming to the generalizability of the findings. Future work will aim to overcome this limitation by adopting an approach similar to that in [23], exploiting multiple publicly available datasets in the literature to train more generalizable models and enhance the machine learning analysis.

## 6. Conclusions

In this work, we have demonstrated that nuanced levels of stress can be objectively characterized using physiological signals, moving beyond the binary rest-versus-task paradigms that dominate much of the current literature on the topic (RQ1). Importantly, we achieved this discrimination using short analysis windows of around 30 s, with good classification results (RQ2), highlighting the potential for real-time monitoring in interactive and applied contexts. Furthermore, our exploration of influencing factors revealed that baseline stress levels, scored with PSS, modulated the EDA response, suggesting that EDA dynamics may serve as a low-cost marker of vulnerability to chronic stress (RQ3). While this interpretation requires confirmation through larger and more targeted studies, our findings point toward an underexplored dimension of inter-individual variability in stress physiology. Taken together, the presented results support the feasibility of using lightweight, off-the-shelf systems for fine-grained stress monitoring and motivate further research into their applications in personalized health and adaptive human–computer interaction. 

## Figures and Tables

**Table 1:** Description of the computed features. EDA refers to the filtered, windowed, z-scored EDA signal; cvxT and cvxP refer to the *t* and *r* components obtained from the cvxEDA algorithm.

Feature Name	Description
$EDA_{Mean}$/$CvxT_{Mean}$/$CvxP_{Mean}$	Mean of the signal segment
$EDA_{Std}$/$CvxT_{Std}$/$CvxP_{Std}$	Standard deviation of the signal segment
$EDA_{Median}$/$CvxT_{Median}$/$CvxP_{Median}$	Median of the signal segment
$EDA_{Kurt}$/$CvxT_{Kurt}$/$CvxP_{Kurt}$	Kurtosis of the signal segment
$EDA_{Skew}$/$CvxT_{Skew}$/$CvxP_{Skew}$	Skewness of the signal segments
$EDA_{Range}$/$CvxT_{Range}$/$CvxP_{Range}$	Range of the signal segments
$EDA_{max}$/$cvxT_{max}$/$cvxP_{max}$	Maximum value of the signal segments
$EDA_{PB5}$/$cvxP_{PB5}$	Percentage of power in the 0.05–0.5 band
$EDA_{P90}$/$cvxP_{P90}$	Frequency corresponding to the 90% of the spectral power
$EDA_{Pmax}$/$cvxP_{Pmax}$	Frequency corresponding to the max of the spectral power
$EDA_{Pstd}$/$cvxP_{Pstd}$	Standard deviation of the spectral power
$Der_{Mean}$	Mean absolute derivative of EDA segments
$Der_{max}$	Maximum derivative of EDA segments
$NegDer_{Mean}$	Mean negative derivative of EDA segments
$EDA_{Activity}$	Hjorth activity–variance computed on EDA segments
$EDA_{Mobility}$	Hjorth mobility–square root of the variance of the derivative divided by activity of the EDA segment
$EDA_{En}$	Shannon entropy of EDA signal segments
$EDA_{apEn}$	Approximate entropy of EDA signal segments
$EDA_{saEn}$	Sample entropy of EDA signal segments
$EDA_{lyapExp}$	Lyapunov exponent of EDA signal segments

**Table 2:** This table summarizes the statistical repeated-measures ANOVA analysis for the significant features. ANOVA F statistic, generalized eta-squared ng2, and *p*-value (*p*) are shown. Also, post hoc analyses with Bonferroni correction are reported as post hoc significant number of couples (PHC) and post hoc pairs (PostHoc_Pairs). In case of non-normal features, the Friedman non-parametric alternative to ANOVA is reported. In this case, Q represents the Friedman statistic and W represents Kendall’s W. Red: *p* < 0.05, orange: *p* < 0.01, green: *p* < 0.01.

Feature	Test	W/ng2	Q/F	*p*	PHC	PostHoc_Pairs
$oo_{sd1/sd2}$	Friedman	0.15	10.57	0.014	0	
$oo_{pnn50}$	Friedman	0.15	10.57	0.014	2	1-3, 1-4
$oo_{breathingrate}$	ANOVA	0.08	2.88	0.042	1	2-4
$EDA_{Mobility}$	Friedman	0.21	14.22	0.002	2	1-3, 1-4
$cvxP_{Max}$	Friedman	0.64	43.96	<10^−8^	6	1-2, 1-3, 1-4, 2-3, 2-4, 3-4
$cvxT_{Range}$	Friedman	0.25	17.19	0.0006	4	1-3, 1-4, 2-3, 2-4
$cvxT_{Max}$	Friedman	0.92	63.16	<10^−12^	6	1-2, 1-3, 1-4, 2-3, 2-4, 3-4

**Table 3:** Results of LMMs for performance effect, reporting coefficients and their significance for the features. The model is aware of the level, but no interaction term is considered, since this is the formulation Feature∼Game(LABEL)+Performance+(1|SubjectID). Red: *p* < 0.05, orange: *p* < 0.01.

Feature	p_unc	p_Bonf	p_fdr	Coef.
$EDA_{Range}$	0.008	0.281	0.140	−2.0569
$EDA_{PB5}$	0.002	0.083	0.083	95.557
$EDA_{Pstd}$	0.029	1	0.33	0.065157

**Table 4:** Significant correlation coefficients—Pearson ($P_{r}$) and Spearman ($S_{rho}$)—between features and performance, averaged across game levels; * *p* < 0.05.

Coef.	${\mathrm{pp}}_{\mathrm{sd}1/\mathrm{sd}2}$	${\mathrm{pp}}_{\mathrm{breathRate}}$	${\mathrm{EDA}}_{\mathrm{Kurt}}$
P*_r_*	0.332	0.440 *	−0.342
$S_{rho}$	0.448 *	0.532 *	−0.432 *

**Table 5:** Significant correlation coefficients—Pearson (P*_r_*) and Spearman ($S_{rho}$)—between features and PSS score, across all games; * *p* < 0.05, ** *p* < 0.01.

Coef.	${\mathrm{EDA}}_{\mathrm{Range}}$	${\mathrm{NegDer}}_{\mathrm{Mean}}$	${\mathrm{EDA}}_{\mathrm{saEn}}$	${\mathrm{EDA}}_{\mathrm{lyapExp}}$	${\mathrm{CvxT}}_{\mathrm{Mean}}$	${\mathrm{cxvP}}_{P90}$
P*_r_*	−0.501 *	0.524 *	0.552 **	0.580 **	0.463 *	0.426 *
$S_{rho}$	−0.545 **	0.618 **	0.295	0.538 **	0.448 *	0.425 *

## Data Availability

The dataset containing the features computed is available in the GDD_EDAHRV repository, along with generated sub-datasets and the code to produce the results. Raw data are available on request.
